# Implementation of national policies and interventions (*WHO Best Buys*) for non-communicable disease prevention and control in Ghana: a mixed methods analysis

**DOI:** 10.1186/s12961-024-01242-3

**Published:** 2024-11-15

**Authors:** Leonard Baatiema, Olutobi Adekunle Sanuade, Irene Akwo Kretchy, Lydia Okoibhole, Sandra Boatemaa Kushitor, Hassan Haghparast-Bidgoli, Raphael Baffour Awuah, Samuel Amon, Sedzro Kojo Mensah, Carlos S. Grijalva-Eternod, Kafui Adjaye-Gbewonyo, Publa Antwi, Hannah Maria Jennings, Daniel Kojo Arhinful, Moses Aikins, Kwadwo Koram, Ann Blandford, Edward Fottrell

**Affiliations:** 1 Department of Health Policy, Planning and Management, School of Public Health, University of Ghana, P.O. Box LG 13, Accra, Legon Ghana; 2 Centre for Tropical Medicine and Global Health Research, Nuffield Department of Medicine, University of Oxford, Oxford, United Kingdom; 3 Department of Population Health Sciences, Division of Health System Innovation and Research, Spencer Fox Eccles School of Medicine at the University of Utah, Salt Lake City, United States of America; 4 Department of Pharmacy Practice and Clinical Pharmacy, School of Pharmacy, University of Ghana, Accra, Ghana; 5 Institute for Global Health, University College London, London, United Kingdom; 6 Department of Community Health, Ensign Global College, Kpong, Ghana; 7 Department of Food Science and Centre for Sustainability Studies, Stellenbosch University, Stellenbosch, South Africa; 8 Noguchi Memorial Institute for Medical Research, University of Ghana, Accra, Ghana; 9 London School of Hygiene & Tropical Medicine, London, United Kingdom; 10 Institute for Lifecourse Development, University of Greenwich, London, United Kingdom; 11 Mental Health and Addictions Research Group, Department of Health Sciences, University of York, York, United Kingdom; 12 Department of Computer Science, University College London, London, United Kingdom

**Keywords:** Non-communicable diseases, Implementation, WHO Best Buys, Policy, Barriers, Ghana

## Abstract

**Background:**

The World Health Organization (WHO) encourages all member states to adopt and implement a package of essential evidence-based interventions called the Best Buys to reduce the burden of non-communicable diseases (NCDs). To date, little is known about the implementation of national policies and interventions for NCD control in the WHO member states in sub-Saharan Africa. Our study aimed to evaluate the implementation of national policies and interventions (*WHO Best Buys*) for non-communicable disease prevention and control in Ghana.

**Methods:**

This was explanatory mixed methods research which started with a document review of Ghana’s WHO Best Buys scores from the 2015, 2017, 2018, 2020 and 2022 WHO NCD Progress Monitor Reports. Thereafter, we conducted 25 key informant interviews and one focus group discussion (11 participants) with key policymakers and stakeholders in the NCD landscape in Ghana to understand the implementation of the NCD policies and interventions, and the policy implementation gaps and challenges faced. Data from the NCD Progress reports were presented using mean scores whilst the qualitative data was analysed thematically.

**Results:**

Ghana has shown some advancements in the implementation of the WHO Best Buys measures. Ghana’s implementation scores for 2015, 2017, 2020 and 2022 were 5.0, 9.0, 5.0 and 5.5 respectively, against the mean implementation scores of 7.6/19 for lower-middle-income countries and 9.5/19 for upper-middle-income countries. Efforts to decrease major risk factors such as excessive alcohol consumption and unhealthy diet have been progressing slowly. The most common challenges were related to a) the role of socio-cultural factors, b) stakeholder engagement, c) enforcement and implementation of public health policies, d) implementation guidelines, e) public awareness and education on NCDs, f) financing of NCD prevention and control, g) curative-centered health systems, and h) over-centralization of NCD care.

**Conclusion:**

Ghana has made progress in adopting the WHO Best Buys targeting risk factors of NCDs. However, the country faces contextual barriers to effective implementation. With the retrogression of some measures over time despite making progress in some earlier years, further investigation is needed to identify facilitators for sustained implementation of the WHO Best Buys interventions.

**Supplementary Information:**

The online version contains supplementary material available at 10.1186/s12961-024-01242-3.

## Introduction

Non-communicable diseases (NCDs) are now a major public health challenge globally. Earlier estimates reported that NCDs are the leading causes of mortality, accounting for 41 million deaths annually, with 77% of these mostly premature and avoidable deaths occurring in low- and middle-income countries (LMICs[1]). To date, evidence from the International Diabetes Federation suggests that an estimated 451 million people were living with diabetes in 2017 globally and this is projected to increase to about 693 million by 2045, primarily precipitated by aging populations and lifestyle changes [2]. About 80% of the global NCD burden is now reported from sub-Saharan Africa (SSA) and other LMICs [3–7]. Recent evidence suggests that the increasing burden of NCDs in SSA and LMICs in general is attributed to an increase in demographic, nutritional, sociocultural, and economic transitions [2, 5, 8–11]. This is further compounded by the fact that awareness of NCD risk factors is quite low [8, 12, 13] access to screening and management services is also poor [13–16] and a significant proportion of the people are undiagnosed [8, 12, 14, 17].

In recognition of this public health challenge, the World Health Organization (WHO) has encouraged all member states to adopt and implement a package of essential evidence-based interventions called Best Buys to reduce the NCD burden. These Best Buys focus on addressing the primary causes of NCDs, including tobacco use, unhealthy diets, alcohol use, and lack of physical activity. They are extremely cost-effective and provide substantial returns on investment for governments that choose to implement them. Additionally, the implementation of these Best Buys interventions has been shown to prevent premature deaths from NCDs [18–20]. Policy responses, investments, and plans to prevent and treat NCDs vary from one context to the other [12], and there is a substantial gap in access and availability of care for NCDs across different countries and regions of the world [21]. Evidence on implementation of the Best Buys policies in SSA is limited [22]. Similarly, the evidence on the impact of plans, interventions, and policies to mitigate the NCDs’ consequences or prevent them is scarce in the region [12, 23]. The slow pace of implementation of the WHO Best Buys is in part due to the limited contextualization of the Best Buys interventions, particularly contextual factors that have impeded the implementation process [24]. For example, evidence from seven Asian countries recently showed that factors such as funding, poor multisectoral collaboration, limited organizational capacity, low awareness of NCD risk factors, and low political will were among the key challenges hampering the implementation of the WHO Best Buys [25].

In Ghana, NCDs have surged over the past decade, accompanied by an increase in exposure to risk factors. Based on recent estimates, about 43% of the overall deaths in Ghana are attributed to NCDs [26]. Public awareness of NCDs and the associated risk factors remains low [27, 28], and could be as low as 18% in a population of people living with hypertension [29]. At the community level, NCDs such as diabetes are socially represented diversely, and this tends to influence the health-seeking behaviours of people in accessing NCD care [30]. Out-of-pocket payments for NCDs remain quite high despite the implementation of the National Health Insurance Scheme which was introduced to replace the cash-and-carry payment mechanism [31] A recent study that examined the economic burden of type-2 diabetes (T2D) and hypertension comorbidity management found that a quarter of the respondents pay for their healthcare through co-payment and insurance jointly, and 42.9% pay out-of-pocket (OOP), and where those within the lower wealth quintile spent a lot more relative to those in the higher wealth quintile [32].

Against this background, there have been several efforts to develop programs and policies for the prevention and control of NCDs. These efforts encompassed the creation of a national NCD prevention and control programme, as well as the formulation and update of this national policy in both 2016 and 2022, respectively. Owing to the high political will for NCDs, the country recently hosted the International Strategic Dialogue on NCDs with the development of a global compact to guide efforts towards NCD prevention and control worldwide. Despite these efforts, the burden of NCDs continues to witness an astronomical rise; out-of-pocket payment for the management of NCDs is also high, and there is sub-optimal access to screening, diagnosis, and treatment services [33, 34]. To the best of our knowledge, no scientific evaluation has been conducted to assess the Best Buys implementation status and barriers to their intervention’s implementation in Ghana, despite Ghana's commitment to the Global Action Plan on NCD prevention and control. Our study provides valuable first-hand, foundational information on Ghana's status regarding the implementation of the WHO Best Buys interventions.

## Methods

### Study design

An explanatory sequential mixed-methods design was employed—firstly, through a quantitative assessment of Ghana’s WHO Best Buys scores from the 2015, 2017, 2020, and 2022 WHO NCD Progress Monitor Reports. Secondly, through key informant interviews of policymakers and stakeholders in the NCD landscape in Ghana, to understand the implementation of the NCD policies and interventions, and the policy implementation gaps and challenges faced. This paper is part of the Contextual Awareness, Response and Evaluation (CARE) Diabetes in Ghana project which aims to generate data to understand the burden, narratives, socioecological drivers, consequences, and responses to diabetes in Ga Mashie. In addition, it aims to explore opportunities for community-based interventions for diabetes prevention and control in Ga Mashie.

### Study population and recruitment

In this study, we interviewed healthcare managers/providers, policymakers, and experts involved in the prevention and control of NCDs in Ghana. We specifically targeted community opinion leaders, policy implementers, policy makers (from the Ghana Health Service—GHS and the Ministry of Health (MoH), policy influencers (e.g., media outlets and social media, Civil Society Organisations (CSOs), and industry bodies), NCD Patient Advocates, and relevant stakeholders in the NCDs space to examine the implementation of key NCD policies and interventions within the context of the WHO Best Buys. Researchers, the media, CSOs, and development partners such as USAID, WHO, JICA and PATH Ghana were also approached. This category of participants was targeted due to their in-depth and comprehensive understanding of NCDs and the major risk factors and their in-depth knowledge about the implementation challenges associated with programs for the prevention and control of NCDs in Ghana. The recruitment approach adopted both formal and informal procedures. For instance, unit heads and administrators were approached with formal letters of introduction, information sheets and requests for participants.

### Sampling procedure

We employed a purposive sampling technique to recruit a diversity of participants from the GHS, MoH, the Ministry of Food and Agriculture, the Ministry of Finance, and the Ghana Revenue Authority, the Food and Drugs Authority, the Ministry of Trade and Industry, and CSOs/NGOs. Recruitment and interviews were conducted until a point of saturation was reached [35]. Overall, 25 key-informant interviews and one focus group discussion (5 females and 6 males) were conducted to effectively capture the full range of participants’ experiences [35].

### Data collection

For the quantitative assessment, we downloaded all the NCD Monitor Reports for Ghana from 2015, 2017, 2020, and 2022. Using the NCD Progress Monitor criteria, evidence of the policy/intervention implementation status was recorded or extracted to highlight full, partial, or no implementation of the policies. Detailed information on the WHO Best Buys, the definition of the interventions and country level targets and the indicators for achievements are outlined in Supplementary File 1, generated from the primary WHO source document [36].

On the other hand, for the qualitative data collection, Key Informant Interviews (KIIs) and a focus group discussion (FGD) were conducted with the aid of a topic guide developed by the research team based on the thematic areas of interest. The data collection was conducted from November to December 2022. The topic guide obtained data on the implementation of policies to control NCD risk factors about the restriction, regulation, and taxation of harmful products and general issues around the state of implementation of the WHO Best Buys policies and interventions. Participants were also asked about the barriers to the implementation of the policies and WHO Best Buys interventions. Data collection was done in English by trained postgraduate students in public health (one male and three females) who had prior experience with qualitative data collection and were well conversant with the study context and subject. All interviews were digitally recorded with participants' consent obtained before the interviews. On average, the KII interviews lasted for about 45 min whilst the FGD was about 1 h.

### Data analysis

The quantitative assessment of the Ghana reports from the WHO NCD Monitor was reviewed and scores were extracted according to the reporting year noting full implementation, partial or no implementation. The level of implementation of the policy based on 2015, 2017, 2020, and 2022 reports was recorded as 1 for full implementation, 0.5 for partial implementation, and 0 for no implementation or when data were missing. Subsequently, we created heatmaps to illustrate the implementation status of each of the WHO Best Buys policies.

All interviews were transcribed verbatim by trained fieldworkers who also conducted/facilitated the interviews. Transcripts were analysed thematically using the framework approach [3]. Analysis iterated between the data and the initial topic guide to assess whether the a priori themes were in the data and whether additional themes needed to be added to the coding frame [4]. All transcripts were imported into QSR NVivo 12 software to facilitate data coding, analysis, and reporting. Data are reported following the Consolidated Criteria for Reporting Qualitative Research (COREQ) guidelines [37].

### Ethics

This study obtained ethics clearance from the Ghana Health Service Ethics Committee (Protocol ID#: GHS-ERC 017/02/22) and the University College London Research Ethics Committee (Study ID # 21541/001). The protocol was also reviewed by the Noguchi Memorial Institute for Medica Research Institutional Review Board (NMIMR-IRB CPN 060/21-22 IORG 000908). Informed consent was obtained and recorded from all individuals before participation in the study. All interviews (audio recordings and transcripts) were anonymized and stored on an encrypted password-protected USB flash drive.

## Results

### Implementation status of the WHO Best Buys for NCD Prevention and Control in Ghana

#### Quantitative findings

Since 2015, Ghana has made progress in implementing the WHO Best Buys measures aimed at reducing the burden of NCDs. Based on the WHO progress monitors, Table 1 displays how Ghana has fared over the four waves of monitoring the implementation of the WHO Best Buys interventions. Globally, the mean implementation scores for lower-middle-income countries and upper-middle-income countries are 7.6/19 and 9.5/19 respectively [25], whereas Ghana’s mean implementation scores for 2015, 2017, 2020, and 2022 were 5.0, 9.0, 5.0 and 5.5 respectively.

**Table 1:** Demographic characteristics of the study participants

Demographics	Number	Percentage
Sex		
Male	16	44.4
Female	20	55.6
Data collection type		
IDI	25	69.4
FGD	11	30.6
Affiliations		
Ghana Health Service Officials	6	16.7
Ministry of Health (policy) Officials	4	11.1
Development Partners (NGOs)	5	13.9
CSOs/NCD Patient Advocates	8	22.2
NCDs Specialists/Consultants/Service providers	7	19.4
Academic/Researchers	2	5.6
Media	1	2.8
People with Lived Experiences of NCDs	3	8.3
Years of professional experience		
1–3	2	5.5
3–6	4	11.1
7–10	10	27.8
11–14	8	22.2
15 and above	12	33.3

An analysis from the NCD Progress Monitor Reports revealed that although for some indicators, Ghana recorded some progress in the implementation of the WHO Best Buys interventions, there are still gaps as illustrated below.

#### Targets, data collection, and plans

In terms of meeting national NCD targets, Ghana performed poorly by not achieving targets in 2017, 2020 and 2022 except for 2015 where there was no response. The collection of routine mortality data was also not achieved. However, Ghana achieved partial implementation of regular risk factor surveys in 2015, 2017, 2020 and 2022. The multisectoral NCD plan was only achieved in 2015 and 2017.

#### Tobacco demand-reduction measures

Regarding tobacco demand-reduction measures, Ghana fully and partially achieved some of the measures. For example, bans on advertising, promotion, and sponsorship were fully achieved. Large graphic health warnings/plain packaging were partially achieved. Mass media campaigns only saw full achievement in 2022. However, measures such as increased excise taxes and prices, and smoking-free policies were partially or not fully implemented. While smoking-free policies were partially achieved in 2017 and 2020, increased excise taxes and prices have not been achieved.

#### Harmful use of alcohol reduction measures

In 2015 and 2017, there was a partial or full achievement on all three measures: restrictions on physical availability, advertising bans or comprehensive restrictions and increased excise taxes. However, in 2020 and 2022, physical availability, advertising bans, or comprehensive restrictions were not achieved and there was no response to increased excise taxes.

#### Unhealthy diet reduction

Ghana has not been successful at addressing the Best Buys measures for unhealthy diet reduction, except for the marketing of breast milk substitute restrictions, where this measure was fully achieved in 2017, 2020 and 2022. From 2015 to 2022, measures aimed at reducing unhealthy diets have not been successful in implementing policies on salt/sodium intake, restricting marketing targeted at children, and policies on saturated fatty acids and trans-fats.

#### Public education and awareness campaigns on physical activity

Ghana achieved Best Buys measures related to public education and awareness campaigns on physical activity in 2015 and 2017, but not in 2020 and 2022.

*Guidelines for the management of cancer, cardiovascular diseases (CVD), diabetes, and chronic obstructive pulmonary disease (COPD),*

Except in 2015, Ghana has fully achieved guidelines for the management of cancer, CVD, diabetes, and CRD, which are essential in improving the management and treatment of NCDs.

#### Drug therapy/counselling

Ghana was not able to achieve drug therapy/counselling to prevent heart attacks and strokes in 2017 and 2022, with no data available in 2015, and no information provided in 2020.

### Characteristics of participants in the interviews and focus group discussions

Overall, 36 participants were recruited and interviewed through 25 KIIs and 1 Focus Group Discussion. Participants were drawn from different disciplines, occupations, and institutions relevant to NCD policy formulation, advocacy, service provision, policy influencers, research, and people with lived experiences of NCDs. This was done to ensure diversity and richness in perspectives about NCD policy and intervention implementation in Ghana. for example, participants from the GHS and the Ministry of Health (MoH), policy influencers (e.g., media outlets and social media, Civil Society Organisations (CSOs), and industry bodies), NCD Patient Advocates, and relevant stakeholders in the NCDs space were recruited. Development partners such as USAID, WHO, JICA and PATH Ghana were also approached and interviewed. Table 2 provides further details of the study participants.

**Table 2:**
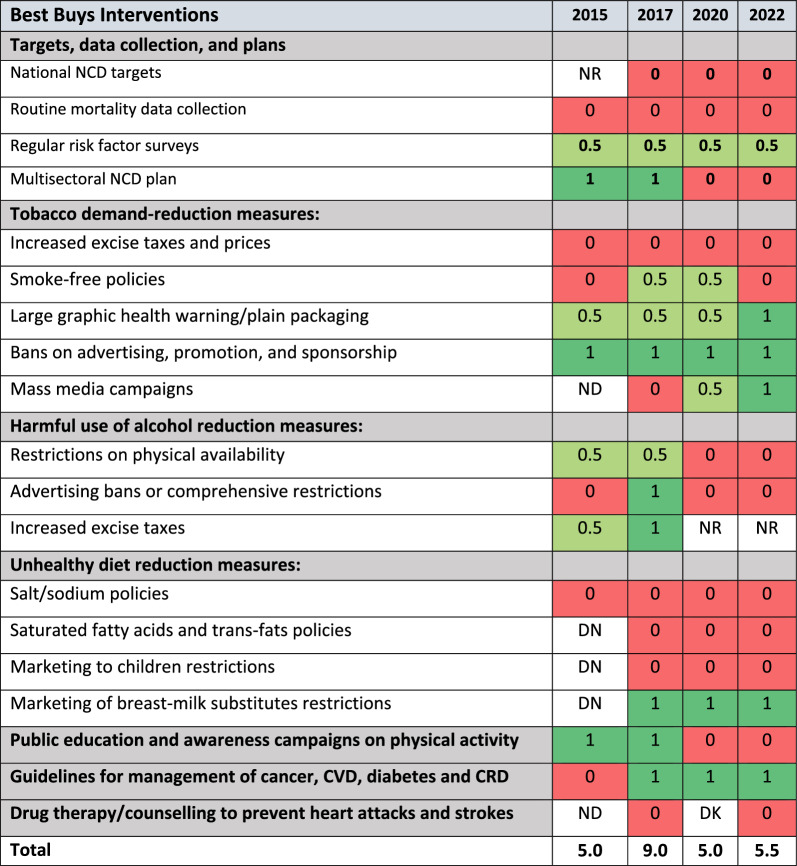
WHO Best Buys Implementation in Ghana

Fully achieved = 1, Partially achieved = 0.5, Not Achieved = 0, Don’t Know = DN, No Data = ND, No Response = NR. Note: Deep green represents full implementation, = 1 point; Light green represents partial implementation = 0.5 points, and Red represents no implementation = 0; the white represents no data or no response

### Barriers to the implementation of policies and interventions for NCD prevention and control

#### Summary of themes

This section reports findings from an analysis of key informant interviews and focus group discussions with policymakers, policy influencers and implementors about the potential barriers to the implementation of the WHO Best Buys interventions to prevent and manage NCDs in Ghana. Overall, eight main themes were identified, and these comprised (a) stakeholder engagement (b) enforcement of public health policies (d) public awareness and education on NCDs and risk factors (e) underfinancing and lack of pooled funding (f) curative-centred health systems, (g) over-centralization of NCD care and (h) socio-cultural context.

##### Theme 1: Socio-cultural context

Participants identified socio-cultural factors as major barriers to the implementation of some of the WHO Best Buys for NCD risk prevention or management. Some participants believed that cultural and social practices are one of the reasons why it is difficult to control alcohol use. For example, participants expressed concern about socio-cultural practices such as weddings, festivals, funerals, and other social events where alcohol is seen as one of the key forms of presents or gifts. Such cultural practices, according to participants, act to promote the sale and use of alcohol and are seen as counterproductive to the measures and policies that seek to prevent high intake of alcohol.

*“That goes deeper into the community because people’s mentality, cultural beliefs, their cultural mindset prevent the implementation of policies. For instance, you know how in our cultural settings, people take the glory when someone presents alcohol during weddings, funeral rites, etc. So, you educate someone on alcohol consumption, they will tell you alcohol is good, even our forefathers consumed it.” Focus Group Discussion, CSO Representative*

Some participants expressed doubt about how existing efforts to control excessive alcohol intake will have any material effect on alcohol intake since this was believed to be part of the societal fabric.

*“The thing is that it is our culture. Right from birth to death or the passage of rites, alcohol is involved. There’s barely any festival in this country where you’ll not see alcohol or a major alcoholic company sponsoring it.” KII, NCD Patient Advocate*

The socio-cultural interpretation of body image was also noted to be a major barrier to efforts to ensure people eat and live healthy lives. Central to this point was a discussion about the social representation of obesity and overweight which is seen in certain contexts as a sign of wealth and good life. Such notions influence what people eat and the kind of body image they want to be associated with. They believe these societal preferences tend to underline public health interventions that seek to create awareness about NCD risk factors and preventive activities.

##### Theme 2: stakeholder engagement

Another theme that emerged from both the interviews and FGDs was the lack of stakeholder engagement and partnership to design and implement programs and policies toward the prevention and control of NCDs. Although stakeholder partnership and the whole-of-government approach were emphasized for NCD prevention and control, participants especially those from the CSO, NGOs and the non-health sector institutions expressed concern about the limited or lack of space for engagement with policymakers in the design, review, and implementation of NCDs policies and programmes.

*“For instance, in the recent national NCD control policy, there was very limited engagement with the non-state actors. I was engaged a bit and to an extent. However, at some level, I wasn’t aware of what was happening despite national and global recognition of our work in the NCD space. We have worked with some facilities across the country, which is why I said, we serve as a bridge between patients’ groups, and the care providers in facilities yet when it comes to the core business of designing or reviewing NCDs policies and programmes, we play second fiddle.” KII, *CSO Representative

The private sector, mainly private health care providers, and business owners such as importers were notable groups cited by participants as another group believed to be less involved in the development and implementation of policies for NCD prevention and control. Although this generated a mixed reaction among participants since some groups were benefiting from the sale of ultra-fat products, tobacco, and alcohol, the point was made for them to be included in discussions and implementation of programs to curtail or regulate the importation and sale of products that tended to promote NCDs risk. Involving these stakeholders was noted to be essential to ensure all interest groups were included in NCD policy deliberations.

*“If you consult and you have buy-in, the implementation is easier. If you can identify the right stakeholders, then implementation is earlier. So one of the issues is how the evidence is generated. The plan that, this stage who are the people that the evidence is intended to help, and do they feel a part of it, a part of creating it, was there an implementation plan right from the beginning that if we find something, this is our implementation strategy, these are stakeholders - important people or place that we need to engage and get buy-in right from the beginning, so that we can implement.” FDG, private health sector participant”*

##### Theme 3: Enforcement of public health policies

A common theme that arose from the key informant interviews relates to the weak enforcement of laws and regulatory frameworks for NCD prevention, especially regulatory frameworks on the use of tobacco. Ghana also developed and implemented different regulatory frameworks, policies, and laws to reduce population exposure to NCD risk factors, ban on public smoking, designated smoking rooms (DSR), ban on sponsorship and advertisement on the radio, sponsorship of events, television, and billboards to promote tobacco and alcoholic beverages. Despite this, enforcement of such initiatives and regulatory frameworks has been a major challenge.

*“If I could add to her point, I think the challenge has been our enforcement. We have the laws, but enforcement has always been a challenge. Just like she's said, the selling to underage children. These days, you see a 15-year-old and he looks like yourself. In other jurisdictions, for example, I was in the USA and one Friday, I went out to buy alcohol. The lady said that she was not going to sell the alcohol to me unless I showed my ID card. I said, “Look at me, I'm more than 20 years old.” FGD MOH Official,*

Participants conceded that although frameworks and laws existed to restrict access, the tobacco and alcoholic beverage companies were quite aggressive and innovative with their marketing strategies that often seemed to be ahead of the game in facilitating access to these products.

*“The people are even willing to sell to people underage. So, we have beautiful laws, but the enforcement is zero. So, we should enforce the laws. There’s a law that says we shouldn’t sell to people underage. Which of the supermarkets here would you go to buy alcohol and they will check for your ID card before they sell it for you? So, it is a matter of enforcement when it comes to the issues of availability and accessibility. The laws are there but they don’t enforce them. So, we should emphasize that the laws should be enforced.” KII, CSO Representative*

##### Theme 4: Implementation of guidelines

Although participants conceded that implementation and enforcement of existing frameworks, laws, and policies are sometimes weak, slow, or lacking in some cases, they however added that where such policies existed to control or prevent NCDs or the risk of NCDs, implementation guidelines are often lacking or outdated. According to participants, some NCD risk factors such as alcohol have policies but no legal framework or laws to support implementation.

*"No policies or legal frameworks are supporting front-packaging warning labels on canned or packed foods to tackle NCDs in Ghana. We are not there yet. For example, in 2017, we launched the alcohol control policy. We are currently working on a legislative instrument, thus; an alcohol control legislative instrument to give the policy a legal backing the absence of which will limit the mandate of key institutions such as the FDA.” KII, NCD Researcher*

Participants expressed concern about the lack of evidence-based guidelines or up-to-date guidelines to ensure sufficient implementation of activities to counter the aggressive and smart innovations employed by the tobacco and alcohol industries to increase access and use of their products. One key example highlighted by participants was the lack of guidelines for the packaging of alcohol products.

*“Packaging alcohol in these small bags makes or entices children to easily purchase it. So, if we can look at how the alcohol is been packaged and not use that small pack which is been sold for GHC0.50. Which makes it cheaper for people to buy. People easily have access and can afford this package in this smaller bag. So, we can also consider the packaging issue as well.” KII, CSO Representative*

##### Theme 5: Public awareness and education on NCDs and the risk factors

Participants observed that although NCD risk factors were on the rise, awareness at the community or population level was low. Participants attributed the low awareness of NCDs and their risk factors to limited awareness campaigns and activities, education on healthy diets, and the risk of NCDs among those who smoke tobacco. It also came to light that most people were not aware of the major risk factors for NCDs and thus tended to engage in activities that put them at risk of NCDs. One participant cited air pollution or second-hand smoke as a major NCD risk factor that is less known among the populace.

*“From the global burden of diseases that were shared, it was reported that the soot in our atmosphere is very high. People are always burning. If you go into people’s homes, you’ll find them burning easily. They think it is easy but they’re creating dark soot. In fact, Accra is one of the most polluted cities in the world. The air pollution in Accra is really bad and potentially exposing people to different illnesses, including NCDs”. KII, Diabetologist*

Despite the rise in NCDs because of air pollution, participants believed most people have a limited understanding of the major NCD risk factors such as pollution and as a result, they engaged in activities domestically and commercially that predispose them to NCDs and their risk.

##### Theme 6: Underfinancing and pooled funding for NCD care

Participants identified limited funding for NCD prevention and treatment services. This was explored in two dimensions. Firstly, participants observed that funding for NCD prevention and control programmes was limited and as a result, public health interventions to support awareness and education of NCDs and the major risk factors were lacking.

*“It’s easy to say funding might be a challenge but let’s say you want to do screening, you want to educate people, you have to move into the community - how do you move? You are going to need resources. you are asking me about manuals and things like that, all those things involve money. if for example, I say ok let me go to Malata market to talk about diabetes, when I go, I expect that I will be checking their sugar and other things for them. So that’s where the resources come in” KII, Diabetologist.*

On the other hand, according to views from the KIIs and the focus group discussion, a major source of concern for people living with NCDs is the limited pooled funding for NCDs and the high cost of treatment services for NCDs. Given the chronic nature of the conditions, the cost of treatment for NCDs as experienced by participants pushes most into poverty and affects their livelihood and as a result, purchasing medication and other treatment services becomes a challenge, resulting in treatment non-adherence and complications thereof. Here, participants unanimously highlighted the need to review the benefit package for NCDs to ensure access to quality treatment services for NCDs and better patient care.

*“Due to the pill burden, I sometimes default in my treatment or do not adhere to treatment. But the major problem I face regularly is the ability to pay for my medications so when I need to refill and I don’t have money, I just go off medication until I have funds to buy medicines. My medications are not covered by national health insurance, so everything is out of pocket for now. ‘’KII, Person with lived experience of NCDs”*

##### Theme 7: Curative-centred health systems

Participants in the focus group discussion highlighted the skewness of the health systems towards curative care and limited preventive services for NCDs. Their view was supported by their observation that the benefit package for the current National Health Insurance Policy in Ghana does not cover or reimburse claims for preventive services for NCDs. Medications that can prevent further complications, more episodes, or exacerbate other conditions among people with chronic conditions are not covered by the national health insurance scheme and they see this as a major setback to efforts to UHC and SDG attainment by 2030.

*At least you will get insulin on the NHIS, you will get metformin… but you will not get medications which we know can prevent stroke, you won’t get medications which improve kidney outcomes - is not on the NHIS. you won’t get insulin supplies, so you must buy needles, and you won’t get the glucometer so how do you manage when you don’t know what is happening? We need to rethink our health systems and priorities as a nation. For example, diabetes is such that whether the sugar is 20 or 15, the patient may feel the same. Even some drugs on the NHIS, unless you have a result showing what your cholesterol is high and you have to pay to get that cholesterol test done. For me, it does not make any sense because it’s not about the cholesterol level but is about what is the person’s risk, if the cholesterol level is 2 and there is previous CVD, they still need the medicine. if the cholesterol level is 1.7, I still need to drive that down so it’s not about what is the level, but what is the person’s risk, yet these medications don’t get reimbursed, so patients pay from their pockets KII, Diabetologist.*

Participants expressed further concerns about the curative nature of the health systems, and this poses a major challenge to efforts towards the provision of screening and diagnostic services for NCDs. They opined that countries that have made major progress in reducing NCDs burden tackled both the curative and prevention but the Ghanaian health system in their view prioritises curative services.

*And you know the challenge is that we are more interested in cure, cure, cure, cure and not preventive. People think preventive is cheap, but it’s not. In terms of quality prevention, who is going to talk about it? who is going to screen? who is going to pay for all these things? People think prevention is cheaper, but what goes into prevention is not cheap. Can you imagine the quality of life that you have if you were picked early, rather than having diabetes or hypertension affecting all your organs? As for COVID, hypertension was riskier than that. And I think now the service is more driven towards promotion. That is what the service and ministry is now championing, promoting, promoting, promoting and I think it’s catching up and our promotion is becoming stronger. So, the teaching hospitals have now established diabetes clinics, and now the regional hospitals are picking up, so I think with support we will be able to get there. KII, Physician Specialist*

##### Theme 8: Over-centralization of NCD care

NCD care was described as overly centralized in key major hospitals, mostly secondary and tertiary level hospitals. They believed access to high-quality NCD care was limited at the primary health care (PHC) level and wondered if such service could be decentralized to the peripheries.

*Most detected NCDs at the lower PHC levels are referred to the next level of care where somebody can prescribe and get covered by the NHIS but that often creates a barrier to access because the person must travel out of the district, and they often don’t go when referred due to the cost implications. Some end up going but later when the condition would have exacerbated with complications. We need to start decentralizing NCD care by building capacity for a lower cadre of staff to be able to manage NCD at the various PHC levels. This should include prescriptions by such cadre that the NHIS can reimburse later. ……Yes, NCDs such as the Cancers, etc can be referred but the common ones without complications such as hypertension and diabetes which are the commonest should be treated and managed at the various PHC levels. We need to offer training on simple diabetes and hypertension screening and management. This is one of the cost-effective measures for NCD prevention and yet we are not investing a lot at that level let’s begin to move NCD care to the PHC levels. KII, CSO Representative*

## Discussion

In this mixed methods study, we examined how Ghana has implemented the WHO Best Buys interventions to address non-communicable diseases. We identified barriers to implementation and gaps in current policy that need to be addressed urgently to reduce the burden of NCDs in the country. The study collected primary data using both quantitative and qualitative research methods to report the implementation status of the WHO Best Buys interventions and insights on the barriers affecting their implementation respectively. Overall, Ghana’s implementation scores for 2015, 2017, 2020 and 2022 were 5.0, 9.0, 5.0 and 5.5 respectively, while in the same period, the mean implementation scores for lower-middle-income countries and upper-middle-income countries were 7.6/19 and 9.5/19 respectively. The perspectives of key senior managers, policymakers, patient advocates, people living with NCDs, researchers, CSOs and the media were categorized into eight domains of barriers namely a) sociocultural context; b) stakeholder engagement; c) enforcement and implementation of public health policies and guidelines; d) implementation guidelines; d) lack of awareness and public education on NCDs and their risk factors; e) funding for NCD prevention and control; f) curative-centered health systems and g) over-centralization of NCD care.

### Findings compared with previous literature

Our study unearthed several policy gaps in the implementation of the WHO Best Buys for NCD prevention and control. Despite the ratification of the Framework Convention for Tobacco Control [38], stakeholders were of the view that enforcement of policies to restrict access, regulate the sale of tobacco products, stop smoking in public, or develop cessation programmes that were limited or poorly implemented if any. These findings align perfectly with earlier reports suggesting these factors and policy gaps as key barriers to the lack of progress in implementing policies and measures to control access and use of tobacco products in Ghana [38, 39]. Although similar findings have been recorded in Thailand, researchers observed that Thailand has made major strides over the past years in reducing tobacco use by about 25% because of the implementation of stringent measures to curb tobacco access and use [40, 41]. Recent reviews by Allen and colleagues have highlighted a lack of or weak enforcement of tobacco control policies in most LMICs [25]. It is therefore unsurprising when stakeholders in Ghana expressed similar views about the current policy gaps in the implementation of tobacco control policies.

The context for the implementation of policies has often been cited as a key determinant of the success or otherwise of the policy implementation [42, 43]. According to stakeholders in this work, the socio-cultural context is observed to have influenced the implementation of public health education programmes to prevent or control major NCD risk factors such as unhealthy diets and excessive alcohol intake. For example, it was revealed by participants that the Ghanaian context continues to place a premium on alcohol for festivities, and social and cultural events as a means of refreshment and gifts during occasions. This socio-cultural factor, in their view, acts as a potential barrier to public health awareness programmes. This finding supports the view by researchers regarding the need to contextualize the WHO Best Buys interventions for effective implementation to reduce the risk of NCDs and treatment outcomes for people living with NCDs [44].

Physical inactivity remains a principal contributor to the high burden of NCDs in LMICs, Ghana in this context. In this study, it was noted that Ghana achieved Best Buys measures related to public education and awareness campaigns on physical activity in 2015 and 2017, but not in 2020 and 2022″. Although it remained unclear what could have caused the reversal in the successes chalked in achieving this in the previous years, an immediate plausible explanation could be the COVID-19 pandemic which saw most governments and policymakers diverting resources, reprogramming their existing budgets from other equally critical public health threats such as NCDs to COVID-19 prevention and control efforts [45, 46]. As previously argued, most countries in LMICs lack a comprehensive, proven and robust framework to guide budgetary reallocation or resources distribution in times of a pandemic or a crisis and thus it was quite difficult to definitively and explicitly tell the extent to which resource reprogramming for COVID-19 was going to impact other public health priorities [45].

The Global Action Plan for NCD Prevention and Control advocates for a multisectoral approach as a key catalyst for implementing impactful policies and interventions [19]. Multi-stakeholder action has also been identified recently as one of the key principles in NCD prevention and control [47]. However, evidence shows that the lack of stakeholder involvement in the design and implementation of policies to prevent and control NCDs and the associated risk factors persists. Our study made similar observations about the lack of stakeholder involvement in the development and implementation of NCD policies and interventions. This point has also been observed in other contexts [48], where evidence showed poor multi-sectoral engagements to prevent and control NCDs despite evidence showing the need to approach efforts towards NCD prevention and control from a whole-of-government approach. As recently argued, effective policies to reduce the risk or prevent NCDs in a population will require strong multi-stakeholder approaches in the development and implementation of such policies and interventions. This is critical not only for the effective implementation of such interventions but for their sustainability [49]. Therefore, efforts must be bolstered to promote multi-stakeholder engagement as envisioned in the earlier Global Action Plan for NCDs Prevention and Control. That is, barriers to the current policy gaps could be addressed through joint, multi-sectoral collaborations to develop solutions for the implementation of such policies.

Consistent with observations from our study, several studies have also pointed to the lack of sufficient funding allocations to support the implementation of NCD policies and interventions [25, 50, 51]. Findings from our research echoed this point as participants observed the lack of sufficient budgetary allocations to NCD prevention and control programs. The WHO Independent High-Level Commission on NCDs acknowledged the huge funding gap for NCDs and thus advocated for states and governments of the World Health Assembly member states to allocate more funding for NCD-related activities including raising funding from sin or public health taxes (e.g., excise taxes on sugar-sweetened beverages (SSBs), tobacco, alcohol, among others [20]. The issue of limited funding for NCDs is a long-standing global health challenge and yet, as in our study, other studies continue to express worry about the limited nature of funding for NCD prevention and control interventions. Thus, it is important for a new set of conversations and thinking around alternative ways of raising funding to prevent and control NCDs beyond the traditional sources of funding for NCDs. Allen (2016) presents an analysis of alternative funding sources for NCDs such as mobilization and engagement with the private sector, loans, and taxation, among others which could be considered by policymakers and stakeholders in exploring opportunities to bridge the funding gaps for NCDs [52], which was earlier reported to be less than 5% of the overall development assistance for health programs [50].

### Implications

Our study presents insightful evidence and lessons for policymakers and health managers in taking decisive actions toward the prevention and control of NCDs in Ghana. The evidence shows that although Ghana developed and implemented measures over the years, critical gaps remain, and these gaps helped explain why efforts to develop and implement NCD policies and interventions have yielded little results. For example, participants consistently reported limited funding or specialized budgetary allocation for NCD prevention and care. Concerted efforts should thus be made to ensure sufficient allocation of funds is provided for both curative and preventive services for NCDs. As shown in the WHO Best Buys, low-cost but effective interventions now exist so the government needs to prioritize the allocation of funding for such interventions. Sufficient budget provision to cater for both public health programs to raise awareness about NCDs and their risk factors support risk-pooling and implementation of sustainable social insurance policies that promote risk-pooling to minimize or prevent the currently high out-of-pocket payments for NCDs in resource-poor settings including Ghana. Such funding could also support prevention measures including salt reduction, control of alcohol, tobacco use and ultra-processed foods.

Internationally, decentralization of NCD care and preventive services remains a major imperative and this situation is more persuasive in Ghana and other resource-poor settings where NCD care is hospital-centric, and tertiary-based with less investment at the primary care levels. The limited attention on decentralizing NCD can also emerge and this lends credit to earlier views about the need to improve efforts towards the decentralization of NCD care. This remains one of the surest ways of improving access to screening, diagnosis, and treatment services at the lower levels of care. Ghana has witnessed massive investment in its PHC facilities, and we hope that with this evidence, coinciding with global and local efforts including the pilot WHO PEN interventions package in selected districts, greater attention and need would be secured for more investment in NCD prevention and treatment services at the PHC level. This would thus provide the needed impetus for greater and targeted policy reforms to ensure screening for CVDs, cancers, diabetes, and respiratory conditions would be prioritized. Part of these reforms should also focus on ensuring guidelines for implementation of the various sections of the current NCD policy are developed and operationalized. Without such evidence-based and contextualized guidelines, efforts to implement the WHO Best Buys intervention would lag and Ghana would fall short of its commitments to reduce the NCDs burden by three-quarters by 2030.

Although the previous and current NCD policies emphasized intersectoral and multisectoral approaches to fighting NCDs, consistent with the Global Action Plan on NCD prevention and control where countries are encouraged to adopt a whole-of-government approach through multi-sectoral partnerships, stakeholders still observed that this was limited, and attention was needed to magnify and strengthen such efforts. It is thus incumbent on policymakers and key stakeholders to foster such collaborations from the design and conceptualization of programs and local-level interventions, through implementation and evaluation. Harnessing opportunities from the private sector and other non-state actors to make meaningful and germane strides in these efforts should be encouraged.

Weak enforcement or implementation of policies and laws to control tobacco and ban the promotion and marketing of alcohol and ultra-processed foods has remained low despite the adaptation of the Framework Convention on Tobacco Control, enactment of the Public Health Act, and the recent Passage of the Law to increase taxes on Sugar-Sweetened Beverages. In addition, evidence of corporate actors’ penetration, industry interference and influence on policy development and implementation has been observed [53]. Here, we recommend a whole-society approach to strengthening and enforcing the implementation of such policies and laws. A relook of the current engagement of stakeholders and how these policies, laws and programs are implemented should be reviewed to identify lapses and where fault lines exist in the implementation and enforcement processes, these need to be urgently fixed.

There are critical and fundamental questions about the implementation of the WHO Best Buys interventions for NCD prevention [44]. Our study has shed light on this and provided evidence about the implementation of such interventions following which such gap measures can be developed to attenuate the current burden of NCDs when these Best Buy interventions are adopted in Ghana and other similar settings. Evidence from Ghana also shows that the implementation of some of the WHO Best Buys interventions seemed to have deteriorated over time despite making progress in some earlier years, e.g. partial or full achievement in earlier years but none in more recent years. This calls for further interrogation to understand the reasons for this and plausible means for sustaining gains made over the years.

Overall, the findings point to an urgent need to expedite policy actions to support the implementation of the WHO Best Buys for NCDs. This is important if Ghana is poised to meet the NCD-related targets under the SDGs by 2030. Further, COVID-19 likely impacted the progress made in implementing the WHO Best Buys for NCDs, and in some cases, a reversal of progress may have occurred as health resources were skewed towards COVID-19. This requires further research to unravel this. however, policymakers may also be guided by such experiences and now consider developing explicit frameworks to guide resource allocations in times of crisis (e.g. COVID-19). Frameworks exist to guide this exercise including the three-step process by Emanuel and Persad where they emphasized a) elucidating the fundamental ethical values for allocation, b) delineation of priority tiers for scarce resources, and [3] prioritizing to achieve certain fundamental, universal or nationally agreed values [54].

### Study limitations and strengths

This study should be read with some limitations in mind. First, although most of the stakeholders interviewed were key stakeholders in the NCD space in Ghana, their views were not representative of all stakeholders about NCD policy development and implementation. Additionally, the study did not look at the effectiveness of the NCDs policies and interventions but rather documented the views and experiences of key stakeholders. Despite these limitations, certain strengths of the paper also stood out. Our approach enabled the first-ever, in-depth, and comprehensive analysis of the WHO Best Buys for NCD prevention and control in Ghana, whilst highlighting policy gaps for redress.

## Conclusion

Although Ghana has made major strides in developing and implementing policies and programs to improve access to preventive and treatment services for NCDs, challenges remain. It is therefore important for policy attention to address the identified barriers hampering the implementation of the WHO Best Buys interventions. Multisectoral collaborations are also needed to facilitate efforts towards the implementation of evidence-based interventions. Importantly, the study concludes that funding to support NCD policy implementation and NCD care should be a top priority of the state. Given the chronic nature of these conditions and the potential of NCDs to drive people into poverty and increase the mortality or morbidity burden, it is important for measures to be instituted to support risk pooling for healthcare to minimize out-of-pocket payments. Local-level monitoring of the implementation of the WHO Best Buys is needed as this is critical to ensure persistent prioritization and resource allocation to NCDs care. These should be best practices and lessons learned for countries that have yet to comprehensively review the implementation of the WHO Best Buys for NCD prevention and control.

## Supplementary Information

Additional file 1.

## Data Availability

No datasets were generated or analysed during the current study.
