# Supplementary material for: Implementation of national policies and interventions (WHO Best Buys) for non-communicable disease prevention and control in Ghana: a mixed methods analysis
Source: Health Res Policy Syst. 2024 Nov 15;22:154. doi: 10.1186/s12961-024-01242-3 (PMC11566497; doi:10.1186/s12961-024-01242-3)
Supplement: Supplementary file 1 — Additional file 1. [file 12961_2024_1242_MOESM1_ESM.docx]

**WHO Best Buys, the definition of the interventions and country level targets and the indicators for achievements**

| **Best Buys Interventions Indicator** | **Definition** | **achievement criteria** |
| --- | --- | --- |
| **Targets, data collection, and plans** |  |  |
| National NCD targets | Country has set national NCD targets. The NCD-related targets should be time-bound and based on the 9 voluntary global targets and the WHO Global Monitoring Framework. | - This indicator is considered fully achieved if a country responds “Yes” to the question “Are there a set of time-bound national targets for NCDs based on the 9 voluntary global targets from the WHO Global Monitoring Framework for NCDs?”, and provides the needed supporting documentation. Targets must be time-bound, based on the 9 global targets, and need to address NCD mortality, as well as key risk factors in the country and/or health systems. - This indicator is considered partially achieved if the country responds “Yes” to the question “Are there a set of time-bound national targets for NCDs based on the 9 voluntary global targets from the WHO Global Monitoring Framework for NCDs ?”, but the targets do not cover two of the three areas addressed in the 9 global targets (including mortality) or they are not time-bound. |
| Routine mortality data collection | Country has a vital registration system that captures deaths and the causes of death routinely. The International Form of Medical Certificate of the Cause of Death is completed by certifiers. The International Classification of Diseases (ICD) is used to code the causes of death. The data compiled are made available to policy-makers and researchers.  Data are considered to generate reliable cause specific mortality data on a routine basis if:   - Data from the five most recent reporting years are, on average, at least 70% usable. Usability is calculated as (Completeness (%))*(1- Proportion Garbage) - At least five years of cause-of-death data have been reported to the WHO in the last 10 years.   The most recent year of data reported to the WHO is no more than five years old. | - This indicator is considered fully achieved if the country meets all of the above criteria. - This indicator is considered partially achieved if the country does not meet all of the above criteria but has submitted some vital registration data to WHO. |
| Regular risk factor surveys | Country has completed a STEPS survey or another risk factor survey which includes physical measurements and biochemical assessments covering the key behavioural and metabolic risk factors for NCDs. Country must indicate that survey frequency is at least every 5 years. | - This indicator is considered fully achieved if the country responds “Yes” to each of the following for adults: “Have surveys of risk factors (may be a single RF or multiple) been conducted in your country for all of the following:” “Harmful alcohol use” (optional for the Member States where there is a ban on alcohol), “Physical inactivity”, “Tobacco use”, “Raised blood glucose/diabetes”, “Raised blood pressure/ hypertension”, “Overweight and obesity”, and “Salt / Sodium intake”. For risk factors “Raised blood glucose/diabetes”, “Raised blood pressure/hypertension”, and “Overweight and obesity”, the data must be measured, not self-reported. Additionally, for each risk factor, the country must indicate that the last survey was conducted in the past 5 years (i.e. 2014 or later for the 2019 CCS survey responses) and must respond “Every 1 to 2 years” or “Every 3 to 5 years” to the sub question “How often is the survey conducted?”. The country must also provide the needed supporting documentation - This indicator is considered partially achieved if the country responds that at least 3, but not all, of the above risk factors are covered, or the surveys were conducted more than 5 years ago but less than 10 years ago. |
| Multisectoral NCD plan | Country has a multisectoral, national integrated NCD and risk factor policy/strategy/action plan that addresses the 4 main NCDs (cardiovascular diseases, diabetes, cancer, chronic respiratory disease) and their main risk factors (tobacco use, unhealthy diet, physical inactivity, harmful use of alcohol).  “Multisectoral” refers to engagement with one or more government sectors outside of health. “Operational” refers to a policy, strategy or action plan which is being used and implemented in the country, and has resources and funding available to implement it. | - This indicator is considered fully achieved if the country responds “Yes” to the questions “Does your country have a national NCD policy, strategy or action plan which integrates several NCDs and their risk factors?” and to the subquestion “Is it multisectoral?”. Countries also have to respond “operational” to the subquestion “Indicate its stage” and “Yes” to all of the subquestions pertaining to the 4 main risk factors and 4 main NCDs: “Does it address one or more of the following major risk factors?” “Harmful use of alcohol” (optional for the Member States where there is a ban on alcohol), “Unhealthy diet”, “Physical inactivity”, “tobacco” (all 4 must have “Yes”) and “Does it combine early detection, treatment and care for:” “Cancer”, “Cardiovascular diseases”, “Chronic respiratory diseases” and “Diabetes” (all 4 must have “Yes”). Country must also provide the needed supporting documentation. - This indicator is considered partially achieved if the country responds “Yes” to the questions “Does your country have a national NCD policy, strategy or action plan which integrates several NCDs and their risk factors?” and to the subquestion “ Is it multisectoral?”. Countries also have to respond “operational” to the subquestion “Indicate its stage” and “Yes” to at least two of the 4 main risk factors and at least two of the 4 main NCDs. |
| **Tobacco demand-reduction measures:** |  |  |
| Increased excise taxes and prices | Country has total taxes set at a level that accounts for more than 75% of the retail price of tobacco products. | - This indicator is considered fully achieved if the country has total taxes more than 75% of the price of the most sold brand of cigarettes - This indicator is considered partially achieved if the country has total taxes from 51% up to 75% of the retail price of the most sold brand of cigarettes. |
| Smoke-free policies | Country has all public places completely smoke-free (or at least 90% of the population covered by complete subnational smoke-free legislation).“Completely” means that smoking is not permitted, with no exemptions allowed, except in residences and indoor places that serve as equivalents to long-term residential facilities, such as prisons and long-term health and social care facilities such as psychiatric units and nursing homes. Ventilation and any form of designated smoking rooms and/or areas do not protect from the harms of second-hand tobacco smoke, and the only laws that provide protection are those that result in the complete absence of smoking in all public places. | - This indicator is considered fully achieved if all public places in the country are completely smoke-free (or at least 90% of the population covered by complete subnational smoke-free legislation). - This indicator is considered partially achieved if three to seven public places are completely smoke-free, or the law allows designated smoking rooms with strict technical requirements in five or more places. |
| Large graphic health warning/plain packaging | Country mandates plain/standardized packaging and/or large graphic warnings with all appropriate characteristics. Appropriate characteristics for large graphic warnings include:  • specific health warnings mandated;  • appearing on individual packages as well as on any outside packaging and labelling used in retail sale;  • describing specific harmful effects of tobacco use on health;  • are large, clear, visible and legible (e.g. specific colours and font style and sizes are mandated);  • rotating health warnings and/or messages;  • pictures or pictograms;  • written in (all) the principal language(s) of the country. Appropriate characteristics for plain/standardized packaging include:  • restrictions or prohibitions on the use of logos, colours, brand images or promotional information on packaging other than brand names and product names displayed in a standard colour and font style;  • standardized shape, size and materials of tobacco packaging; and  • no advertising or promotion inside or attached to the package or tobacco product. | - This indicator is considered fully achieved if the country has plain/ standardized packaging and/or large graphic health warnings which are defined as covering on average at least 50% of the front and back of the package with all appropriate characteristics as detailed above. - This indicator is considered partially achieved if there are medium-size warnings, which are defined as covering on average between 30 and 49% of the front and back of package, with some or all appropriate characteristics, or large warnings that are missing some appropriate characteristics. |
| Bans on advertising, promotion, and sponsorship | Country has a ban on all forms of direct and indirect advertising. Direct advertising bans include: national television and radio; local magazines and newspapers; billboards and outdoor advertising; point of sale. Indirect advertising bans include: free distribution of tobacco products in the mail or through other means; promotional discounts; non-tobacco products identified with tobacco brand names (brand stretching); brand names of non-tobacco products used for tobacco products (brand sharing); appearance of tobacco brands (product placement) or tobacco products in television and/or films; and sponsorship (contributions and/or publicity of contributions). | - This indicator is considered fully achieved if the country has a ban on all forms of direct and indirect advertising. - This indicator is considered partially achieved if the country has a ban on national TV, radio and print media, but not on all other forms of direct and/or indirect advertising. |
| Mass media campaigns | Country has implemented a national anti-tobacco mass media campaign designed to support tobacco control, of at least 3 weeks duration with all appropriate characteristics. Appropriate characteristics include:  • campaign was part of a comprehensive tobacco control programme;  • before the campaign, research was undertaken or reviewed to gain a thorough understanding of the target audience;  • campaign communications materials were pre-tested with the target audience and refined in line with campaign objectives;  • air time (radio, television) and/or placement (billboards, print advertising, etc.) was obtained by purchasing or securing it using either the organization’s own internal resources or an external media planner or agency (this information indicates whether the campaign adopted a thorough media planning and buying process to effectively and efficiently reach its target audience);  • the implementing agency worked with journalists to gain publicity or news coverage for the campaign;  • process evaluation was undertaken to assess how effectively the campaign had been implemented;  • an outcome evaluation process was implemented to assess campaign impact;  • the campaign was aired on television and/or radio. | - This indicator is considered fully achieved if the country has a campaign conducted with at least seven appropriate characteristics including airing on television and/or radio. - This indicator is considered partially achieved if the country has a campaign conducted with one to six of the appropriate characteristics. |
| **Harmful use of alcohol reduction measures:** |  |  |
| Restrictions on physical availability | Country has a licensing system or monopoly on retail sales of beer, wine, spirits. Country has restrictions for on-/off-premise sales of beer, wine, spirits regarding hours, days and locations of sales. Country has legal age limits for being sold and served alcoholic beverages. | This indicator is considered fully achieved if:  • a licensing system or monopoly exists on retail sales of beer, wine and spirits;  • restrictions exist for on- and off-premise sales of beer, wine, and spirits regarding hours and locations of sales and restrictions exist for off-premise sales of beer, wine, and spirits regarding days of sales; and  • legal age limits for being sold and served alcoholic beverages are 18 years or above for beer, wine, and spirits. |
|  |  | This indicator is considered partially achieved if there are any, but not all, positive responses to the three indicators above. |
| Advertising bans or comprehensive restrictions | Country has regulatory or co-regulatory frameworks for alcohol advertising through different channels (public service/national TV, commercial/private TV, national radio, local radio, print media, billboards, points of sale, cinema, internet, social media). Country has a detection system for infringements on marketing restrictions. | This indicator is considered fully achieved if:  • restrictions exist on alcohol advertising for beer, wine, and spirits through all channels;  • detection system exists for infringements on marketing restrictions. |
|  |  | This indicator is considered partially achieved if there are restrictions on at least public service/national TV, national radio and billboards but no detection system exists for infringements |
| Increased excise taxes | Country has excise tax on beer, wine, spirits. Country adjusts level of taxation for inflation for alcoholic beverages. | This indicator is considered fully achieved if:  • excise tax on all alcoholic beverages (beer, wine, and spirits) is implemented;  • there are no tax incentives or rebates for production of other alcoholic beverages;  • adjustment of level of taxation for inflation for beer, wine, and spirits is implemented. |
|  |  | This indicator is considered partially achieved if there is excise tax on alcoholic beverages as specified above. |
| **Unhealthy diet reduction measures:** |  |  |
| Salt/sodium policies | Country has implemented national policies to reduce population salt/sodium consumption, including reformulation of food products; establishment of a supportive environment in public institutions to enable lower sodium options to be provided; behaviour change communication and mass media campaigns; and front-of-pack labelling. | - This indicator is considered fully achieved if the country responds “Yes” to the question “Is your country implementing any policies to reduce population salt consumption?” and to the subquestions “Are these targeted at: product reformulation by industry across the food supply; regulation of salt content of food served in specific settings such as hospitals, schools, workplaces; public awareness programme; front-of-pack nutrition labeling? (must have “Yes” to product reformulation by industry across the food supply and/or regulation of salt content of food, and “Yes” to public awareness programme and nutrition labeling”). Country must also provide the needed supporting documentation. - This indicator is considered partially achieved if the country responds “Yes” to the question “Is your country implementing any policies to reduce population salt consumption?”, and “Yes” to at least one of the four subquestions “Are these targeted at: product reformulation by industry across the food supply; regulation of salt content of food served in specific settings such as hospitals, schools, workplaces; public awareness programme; front-of-pack nutrition labeling?”. |
| Saturated fatty acids and trans-fats policies | Country has implemented a policy(ies) to limit saturated fatty acids and virtually eliminate industrially produced trans-fats in the food supply | - This indicator is considered fully achieved if the country responds “Yes” to the questions “Is your country implementing any national policies to reduce population saturated fatty acid intake?” and “Is your country implementing any national policies to eliminate industrially produced trans-fatty acids (i.e. partially hydrogenated oils) in the food supply?”, and provides the needed supporting documentation. - This indicator is considered partially achieved if the country responds “Yes” to either of the aforementioned questions. |
| Marketing to children restrictions | Country has implemented a policy(ies) to reduce the impact on children of marketing of foods and non-alcoholic beverages high in saturated fats, trans-fatty acids, free sugars, or salt. | - This indicator is considered fully achieved if the country responds “Yes” to the question “Is your country implementing any policies to reduce the impact on children of marketing of foods and non-alcoholic beverages high in saturated fats, trans-fatty acids, free sugars, or salt?”, and provides the needed supporting documentation. |
| Marketing of breast-milk substitutes restrictions | Country has implemented legislation/regulations that fully implement the International Code of Marketing of Breast-milk Substitutes. | - This indicator is considered fully achieved if the country is assessed as having national legal measures categorized as “full provisions in law”, whereby countries have enacted legislation or adopted regulations, decrees or other legally binding measures encompassing all or nearly all provisions of the Code and subsequent WHA resolutions. - This indicator is considered partially achieved if the country is assessed as having national legal measures categorized as “many provisions in law” or “few provisions in law”, whereby countries have enacted legislation or adopted regulations, decrees or other legally binding measures encompassing many or few provisions of the Code and subsequent WHA resolutions |
| Public education and awareness campaigns on physical activity | Country has implemented at least one recent (within the past 2 years) national public awareness programme on physical activity. | This indicator is considered fully achieved if the country responds “Yes” to the following question: “Has your country implemented any national public education and awareness campaign on physical activity within the past 2 years?” and supporting documents provide clear evidence demonstrating that one or more of the following activities have been undertaken within the past 2 years:   1. national public-facing mass media education and awareness campaign on physical activity, AND/OR 2. national promotional initiatives supporting a regional or multicountry physical activity (sports) campaigns aimed at increasing awareness and encouraging participation in physical activity (e.g. European Sports Week, Caribbean Wellness Week), AND/OR 3. regular promotional days, held across the year, on physical activity either using the same theme (e.g. “car-free” Sundays) or a physical activity theme is clearly linked with the implementation of multiple health promotion days (e.g. World Heart Day, World Diabetes Day). Undertaking a single promotional day per year is not sufficient to fulfil this criteria.   This indicator is considered partially achieved if the supporting documents provide evidence demonstrating that the country has implemented in the past 2 years one or more community-based initiatives or programmes promoting physical activity and/or increasing access to opportunities for physical activity in community settings (e.g. through schools, parks, workplace, health care) but without any evidence of a public-facing mass media education and awareness campaign. |
| Guidelines for management of cancer, CVD, diabetes and CRD | Government approved evidence-based national guidelines/protocols/ standards for the management (diagnosis and treatment) of the four main NCDs – cardiovascular diseases, diabetes, cancer and chronic respiratory diseases. | - This indicator is considered fully achieved if national guidelines/ protocols/standards exist for all four NCDs (cardiovascular diseases, diabetes, cancer and chronic respiratory diseases), and the country provides the needed supporting documentation. - This indicator is considered partially achieved if the country has guidelines/protocols/standards for at least two of the four NCDs (cardiovascular diseases, diabetes, cancer and chronic respiratory diseases), but not for all four. |
| Drug therapy/counselling to prevent heart attacks and strokes | Country has provision of drug therapy (including glycaemic control for diabetes mellitus and control of hypertension using a total risk approach), and counselling to individuals who have had a heart attack or stroke and to persons with high risk (≥ 30%, or ≥20%) of a fatal and non-fatal cardiovascular event in the next 10 years. | - This indicator is considered fully achieved if the country reports that more than 50% of primary health care facilities are offering cardiovascular risk stratification for the management of patients at high risk for heart attack and stroke and that all drugs listed above were generally available in the primary care facilities of the public health sector. - This indicator is considered partially achieved if the country reports that between 25% to 50% of primary health care facilities are offering cardiovascular risk stratification for the management of patients at high risk for heart attack and stroke and that all of the drugs listed above were generally available in the primary care facilities of the public health sector. |
| Fully achieved=1; Partially achieved =0.5; Not Achieved =0; Don’t Know=DN; No Data=ND; No Response=NR | | |
